# Evaluation of a Community COVID-19 Vaccine Ambassador Train-the-Trainer Program

**DOI:** 10.1007/s10903-023-01502-4

**Published:** 2023-06-05

**Authors:** Alexandra M. S. Corley, Stacey M. Gomes, Keith J. Martin, Sharon Watkins, Kendal Lindsey, Robert W. Frenck, Monica J. Mitchell, Amy R. L. Rule, Lori E. Crosby

**Affiliations:** 1 Division of General and Community Pediatrics, Cincinnati Children’s Hospital Medical Center, 3333 Burnet Ave MLC 7035, Cincinnati, OH 45229 USA; 2 Department of Pediatrics, University of Cincinnati College of Medicine, Cincinnati, OH USA; 3 Division of Behavioral Medicine and Clinical Psychology, Cincinnati Children’s Hospital Medical Center, Cincinnati, OH USA; 4 Center for Clinical and Translational Science and Training, Cincinnati, OH USA; 5 School of Education, University of Cincinnati, Cincinnati, OH USA; 6 Division of General Pediatrics, Johns Hopkins University School of Medicine, Baltimore, MD USA; 7 Department of Pediatrics, Johns Hopkins University School of Medicine, Baltimore, MD USA; 8 Centro SOL-Center for Salud/Health and Opportunity for Latinos, Johns Hopkins University School of Medicine, Baltimore, USA; 9 Community Action Agency, Cincinnati, OH USA; 10 Division of Infectious Diseases, Cincinnati Children’s Hospital Medical Center, Cincinnati, OH USA; 11 Division of Neonatology, Children’s Healthcare of Atlanta, Atlanta, GA USA; 12 Division of Hospital Medicine, Children’s Healthcare of Atlanta, Atlanta, GA USA; 13 Department of Pediatrics, Emory University School of Medicine, Atlanta, GA USA

**Keywords:** COVID-19 vaccinations, Community engagement, Community vaccine ambassador

## Abstract

Racially minoritized groups are more likely to experience COVID-19 vaccine hesitancy and have lower vaccination rates.  As part of a multi-phase community-engaged project, we developed a train-the-trainer program in response to a needs assessment. “Community vaccine ambassadors” were trained to address COVID-19 vaccine hesitancy. We evaluated the program’s feasibility, acceptability, and impact on participant confidence for COVID-19 vaccination conversations. Of the 33 ambassadors trained, 78.8% completed the initial evaluation; nearly all reported gaining knowledge (96.8%) and reported a high confidence with discussing COVID-19 vaccines (93.5%). At two-week follow-up, all respondents reported having a COVID-19 vaccination conversation with someone in their social network, reaching an estimated 134 people. A program that trains community vaccine ambassadors to deliver accurate information about COVID-19 vaccines may be an effective strategy for addressing vaccine hesitancy in racially minoritized communities.

## Background

Vaccination against the novel coronavirus (COVID-19) plays a critical role in reducing the morbidity and mortality caused by the ongoing pandemic. Currently, 67% of the US population is fully vaccinated against COVID-19 [1]. Having friends and family members that are vaccinated is associated with one’s personal decision to vaccinate against COVID-19 [2]. Racially minoritized groups are more likely to experience COVID-19 vaccine hesitancy [3], likely because of past and present-day abuse from healthcare systems and practitioners resulting in mistrust. Compared to their white counterparts, a larger share of Black and Latino/a/x adults reported a desire to “wait and see,” or vaccinate only if required, or expressed “definitely not” planning to get vaccinated against COVID-19 [4]. These beliefs translate into suboptimal vaccination rates and have persisted during the pandemic in the context of high community transmission and the emergence of more deadly and contagious viral variants.

The Centers for Disease Control and Prevention’s (CDC) framework to support vaccination, “Vaccinate with Confidence,*”* identifies the use of trusted messengers as a vital community-based strategy for overcoming vaccine hesitancy [5]. This strategy involves training community members, who may themselves be vaccine recipients, to address specific concerns in priority populations (e.g., adolescents). Previous work indicated that racially marginalized groups may prefer community/religious leaders or friends/family over health professionals as a source of health information about COVID-19 and other vaccines [6, 7]. These community vaccine ambassadors serve as an intermediary between health systems and the larger community and address local barriers to vaccination [8].

Our multidisciplinary team (e.g., hospitals, city health department, Latino/a/x and Black serving community-based health organizations) chose to implement this community vaccine ambassador strategy with the goal of increasing the spread of accurate information about COVID-19 vaccines in the local Black and Latino/a/x communities. Our goal was to ultimately increase COVID-19 vaccination rates. Here, we report on the feasibility, acceptability, and impact of our community COVID-19 vaccine ambassador train-the-trainer pilot. We hypothesized that the program would be feasible and acceptable, and that ambassadors would report confidence in having COVID-19 vaccine-related conversations with others in their community. We set a goal that each ambassador would report having conversations with at least 5 individuals.

## Methods

### Participants and Setting

This program was conducted in Cincinnati, Ohio, a city with a population that is 41% Black and 4% Latino/a/x. The median household income for Hamilton County residents is $42,663. The Latino/a/x population in the Cincinnati metro area grew nearly 50% from 2010 to 2020 [9]. At the end of May 2021, when this program occurred, national statistics showed that 56% of Black adults and 57% of Latinx adults in the US had received at least one dose of a COVID-19 vaccine; this is compared to 65% of white adults [4].

The lead partner on the program was Cincinnati Children’s Hospital Medical Center (CCHMC), a large, free-standing academic pediatric hospital.

### Ambassador Train-the-Trainer Program

Using a 2020 COVID-19 community needs assessment, common vaccine hesitations and myths in both communities were identified. Community stakeholders and the public health education team met to design vaccine education goals and objectives based on hesitancy data, preferred information sources and messengers and identify education modalities to empower community advocates. This work was reviewed and determined to be exempt by the Institutional Review Board at CCHMC (IRB ID# 2020-0465).

We aimed to train at least 25 community residents and people from community-based organizations (e.g., faith-based groups, schools, businesses, community healthcare institutions) with an expressed interest in learning more about COVID-19 vaccination. Recruitment occurred via existing partnerships, word of mouth, organizational/collaborative meetings, and fliers. Interested individuals registered for the event and were emailed a Zoom link for the program.

The 90-minute train-the-trainer program was held in June 2021 and delivered in English with Spanish interpretation available via video. The program included a review of ambassador expectations (i.e., to complete the training evaluation and follow-up survey; to talk with someone about the vaccine following the training). During the training, one Black community member and 1 Latino/a/x community member along with their adolescent children shared their reasons for getting vaccinated. In addition, an infectious disease physician provided information and answered questions about COVID-19 vaccines. Families and experts also addressed common myths identified by community partners. The training ended with talking points for ambassadors, using scenarios for demonstration.

### Measures and Analysis

To assess feasibility, registration and attendance were tracked. Immediately following the program, acceptability (overall usefulness, confidence with conversations) was assessed using a computer-based evaluation survey. Two weeks after the training, ambassadors were emailed a follow-up survey asking for information about any conversations about COVID-19 they had with friends, family, or colleagues since the training. Descriptive statistics summarized quantitative survey data. Open-ended questions were iteratively coded using computer-assisted content analysis completed by authors AMSC and SG. Consensus was reached for code application, and then codes were grouped into categories.

## Results

Of the 41 participants who registered for training, 33 (80%) attended (Table 1). Participants were asked to self-identify their race/ethnicity; 65.6% were Black, 18.8% were white, 12.5% were Latino/a/x and 3.1% were Asian.

**Table 1:** Community COVID-19 vaccine ambassador program ambassador attendee demographics

N = 33 Attendees	
Race (%)	
Black	65.6
Latino/a/x	12.5
White	18.8
Asian	3.1
Affiliation (%)	
Community-based organization member	78.8
Hospital affiliated faculty/staff/student	21.2

Twenty-six attendees (79%) completed the initial evaluation and 17 of 26 (65%) completed the two-week follow-up survey (n = 17) (Table 2).

**Table 2:** Community COVID-19 vaccine ambassador program – feasibility, acceptability and 2-week follow-up

Feasibility			
Registered			41
Attendance (n, %)			33, 80.5%
Completed Evaluation (n, %)			26, 78.8%

Acceptability, (n = 26)		
Theme	%	Demonstrative quote
Increased confidence	93.5	“Overall, training gave me info and confidence to approach people and ask if I could help answer their questions.” [*Nonprofit Project Manager*]
Increased knowledge	96.8	“The training and different medical resources helped me speak to family and friends.” [*Early Childhood Educator*]
Will help address concerns in my community	96.8	“[The] training allowed more knowledge for me to discuss with potential family & friends.” [*Community Services Director*]
Overall Rating (Good or Excellent)	100	“I have a more thorough understanding of how to respond to vaccine hesitancy concerns.” [*Community Health Intern*]
Recommended continuation of program	100	“We must continue to talk and inform. The young people are operating on untrue facts. The program should not stop.” [*Family and Community Partner Coordinator*]

Follow-Up (%) , (n = 17)	
Ambassadors who engaged in conversation with a friends/family member/colleague about COVID-19 vaccination (n, %)	17, 100%
Individuals impacted by training (persons)	
Total	134
Per ambassador	7.9
Ambassadors who engaged in COVID-19 vaccine conversations with…(%)	
Family	88
Friends	65
Colleagues	35
Clients	12
Other	24

For acceptability, all respondents rated the training “good” or “excellent” and would recommend it to others. There was consensus (96.8%) that the content addressed COVID-19 vaccine concerns in their community. Nearly all participants who attended the training reported increased knowledge about COVID-19 and vaccination (96.8%), as well as confidence to have vaccine-related conversations with family and peers (93.5%). At two-week follow-up, all community vaccine ambassadors had engaged in a conversation with a colleague, friend, or family member about COVID-19 vaccination. An estimated 134 people (7.9 people per ambassador) were reached by these follow-up conversations, many of whom planned to get vaccinated following the interaction with their community vaccine ambassador. Family members appeared to be most accessible to trained ambassadors; 88% of our participants had a follow-up conversation with someone in their family within two weeks of our training. Qualitative comments revealed that community vaccine ambassadors would continue to use knowledge and skills they obtained from the training to have COVID-19 vaccine conversations.

## Discussion

This paper describes the evaluation of a train-the-trainer community ambassador program to address COVID-19 vaccine misinformation in Black and Latino/a/x communities. Data indicate that the program was feasible and acceptable. Ambassadors reported increased knowledge and confidence, and felt the information was useful for addressing specific COVID-19 vaccine concerns in their communities.

Two weeks after our training, ambassadors reported having successful conversations about vaccine benefits and risks with approximately 134 people in their social networks. Qualitative data indicated that ambassadors felt confident even if the individuals they educated remained hesitant; ambassadors also felt they were building trust and might influence future COVID-19 vaccine intention. It is notable that most participants recruited as vaccine ambassadors in this study were trusted stakeholders of community-based organizations serving minoritized communities, positioning them as reliable resources based on a previous needs assessment [6]. This data is consistent with a recent qualitative study of Black and Latino/a/x populations demonstrating that receiving factual, consistent, and transparent information from trusted messengers all help to increase trust in the COVID-19 vaccine [10]. A recent systematic review correlated unwillingness to vaccinate against COVID-19 with being Black American [11], indicating a need for interventions such as ours that equitably address community needs.

Limitations of this study include that this was a one-time pilot program with limited recruitment and participation; it is possible that ambassadors who did not complete the follow-up evaluation did not find the program beneficial or did not have conversations in their community. Data was not collected on the age group or gender identity of the ambassadors, further limiting the generalizability of our findings. Future studies with larger sample sizes and a more comprehensive data collection strategy are recommended to determine the impact of this program on vaccination knowledge, intention, and rates in Black and Latino/a/x communities. Future efforts may also allow for collection of additional variables; our work captured myths and misinformation, but there is also room to learn about community strengths and resilience that may facilitate vaccination.

## New Contributions to the Literature

The Community COVID-19 Vaccine Ambassador Program was developed to increase access to trustworthy, accurate information about COVID-19 vaccinations by training trusted messengers to have informed conversations. As evidenced by an increase in knowledge and confidence, the engagement of trusted messengers in this train-the-trainer model holds promise for application of this strategy for addressing COVID-19 vaccination hesitancy in marginalized communities. This community vaccine ambassador model could serve as an archetype for mitigating future challenges during the COVID-19 pandemic and addressing hesitancy with other health behaviors.
